# Deciphering the genomic landscape of Trichophyton indotineae in China and exploring a correlative model for azole resistance prediction

**DOI:** 10.1099/mgen.0.001812

**Published:** 2026-09-16

**Authors:** Heping Xu, Ying Mao, Yixuan Hu, Jiangshan Huang, Wannan Chen, Lingling Lu

**Affiliations:** 1 Department of Laboratory Medicine, Xiamen Key Laboratory of Genetic Testing, The First Aﬃliated Hospital of Xiamen University, Xiamen, Fujian, PR China; 2 Infection Technology Platform, Dian Diagnostics Group Co., Ltd., Hangzhou, 310030, PR China; 3 Department of Infectious Disease, Sir Run Run Shaw Hospital, Zhejiang University School of Medicine, Hangzhou, PR China; 4 Department of Medical Microbiology, Fujian Key Laboratory of Tumor Microbiology, School of Basic Medical Sciences, Fujian Medical University, Fuzhou, Fujian, PR China

**Keywords:** antifungal susceptibility, azole resistance *CYP51B*, dermatophytosis, genomic epidemiology, substitutions in squalene epoxidase (SQLE), terbinafine resistance, *Trichophyton indotineae*

## Abstract

**Background.** *Trichophyton indotineae* is an emerging multidrug-resistant dermatophyte that has rapidly spread across continents, posing a serious threat to global public health. Limited genomic data from mainland China hinder a comprehensive understanding of its evolutionary origin, underscoring the urgent need for molecular susceptibility data to guide clinical antifungal therapy. To clarify the evolutionary origin and genome-wide characteristics of three newly identified clinical isolates of *T. indotineae* from Fujian Province, China, we sequenced these isolates to a mean coverage of 1,300× using the Salus Pro sequencing platform and delineated a comprehensive genomic portrait. Additionally, a linear regression model was constructed between the copy-number variation (CNV) of the *CYP51B* gene and the minimum inhibitory concentration (MIC) values of four antifungal agents to achieve molecular susceptibility prediction for *T. indotineae*. Finally, functional annotation and Kyoto Encyclopedia of Genes and Genomes (KEGG) pathway analyses were employed to identify virulence-associated genes and pathways. Three *T. indotineae* isolates recovered in Xiamen, Fujian Province, China, fell into two distinct phylogenetic clades (Group II and Group IV). Notably, genome-wide scanning identified *CYP51B* CNV and the SQLE p.Phe397Leu mutation as the most prominent genomic determinants associated with azole and terbinafine (TRB) resistance, respectively. Structural modelling provided mechanistic support at the protein level for the functional impact of this mutation, revealing that the SQLE F397 substitution triggers a collapse of the hydrophobic binding pocket, thereby hindering TRB binding. Furthermore, a linear regression model was established to achieve molecular susceptibility prediction, showing a robust correlation between the *CYP51B* CNV and the MIC values of four azoles (*P*<0.05). Core-gene analysis against the reference genome identified a shared core genome. KEGG pathway analysis revealed the biological pathways where virulence genes are located, including the MAPK signalling pathway and the *O*-glycan biosynthesis pathway, which suggests potential pathogenic mechanisms. The study delineates the genomic landscape of *T. indotineae* isolates from China and demonstrates a stepwise elevation in azole MICs. The linear regression model reveals a significant positive correlation between *CYP51B* CNV and MIC values for four azoles, thereby advancing the CNV–resistance association from a qualitative observation to a quantitative prediction. These insights enhance the global understanding of the pathogen’s genomic epidemiology and virulence, establishing a critical foundation for genome-guided surveillance and individualized antifungal therapy.

**Impact Statement**

As an emerging multidrug-resistant dermatophyte, *Trichophyton indotineae* has triggered global public health concerns due to its rapid cross-continental spread; however, limited genomic data from mainland China have long impeded a comprehensive understanding of the molecular basis underlying its drug resistance, evolutionary origin and virulence repertoire. Our study fills this critical gap by presenting the systematic molecular characterization of *T. indotineae* isolates from China, leveraging whole-genome sequencing, phenotypic assays and comparative bioinformatics to address clinically and epidemiologically urgent questions.

## Data Summary

The raw sequencing data and genome files generated in this study have been deposited in NCBI under BioProject accession PRJNA1392850 (https://www.ncbi.nlm.nih.gov/bioproject/PRJNA1392850). And the BioSample accession numbers of the whole-genome sequencing data for TI01, TI02 and TI03 are SAMN54273581, SAMN54273582 and SAMN54273583, respectively. The supplementary data consist of ‘Fig S1.pdf’, ‘Fig S2.pdf’, ‘Fig S3.pdf’ and ‘Fig S4.pdf’, reporting supplementary figures (Figs S1–S4) and ‘Table S1.xlsx’ and ‘Table S2.xlsx’, reporting the supplementary tables (Tables S1 and S2).

All supporting data, code and protocols have been provided within the article or through supplementary data files. Four supplementary figures, one supplementary table are available with the online version of this article.

## Introduction

The emerging dermatophyte *Trichophyton indotineae*, first identified in India, causes tinea of the skin, hair and nails [1–3]. Since 2021, the high-level resistance of Indian isolates to terbinafine (TRB) and azoles has fuelled sustained dermatophytosis outbreaks on various continents globally, with refractory tinea corporis and cruris now reported across Asia [4–8], Europe [9–14] and North America [15–17], evolving into a major global public-health threat. China documented its first imported case of multidrug-resistant *T. indotineae* in 2022 [6]. A retrospective survey at Peking Union Medical College Hospital identified an additional 14 TRB-refractory infections between 2019 and 2023 [18]. During 2022–2024, *T. indotineae* isolates from eastern China converged within phylogenetic Group III, forming a regional clonal cluster [7]. As a major hub for international travel and trade, coastal regions like Fujian Province are at high risk for the introduction of diverse lineages.

Epidemiological studies of *T. indotineae* have consistently linked high-level TRB resistance to five recurrent substitutions in squalene epoxidase (SQLE*,* L393F, L393S, F397L, F415Y and H440Y), with the missense variant Phe397Leu being the most critical [19–21]. Conversely, reduced azole susceptibility in *T. indotineae* is primarily attributed to the overexpression of *CYP51B*(TinCYP51B). This gene encodes lanosterol 14 α-demethylase, the enzymatic target of triazoles – including itraconazole (ITC) and voriconazole (VRC) – within the conserved ergosterol biosynthetic pathway. These genomic alterations can be further categorized into two distinct structural architectures, as initially proposed by Yamada *et al*. [22]. Type I strains are characterized by localized tandem duplications consisting of 2,404 bp end-to-end blocks that encompass only the TinCYP51B locus, representing a highly targeted gene amplification. In contrast, Type II strains harbour significantly larger segmental duplications of 7,374 bp [23]. The reference strain TIMM20114 is widely utilized as a genetic and phenotypic baseline due to its single-copy *CYP51B* status and its well-characterized susceptibility to ITC, VRC and TRB [24]. However, previous studies have often been limited by restricted gene sets, low genomic coverage or a lack of quantitative validation, providing only a fragmented view of the multidrug resistance (MDR) landscape [19–21, 23]. Consequently, the evolutionary origins and genome-wide determinants of the newly emerged *T. indotineae* lineages in China remain unresolved, necessitating an integrated genomic and structural approach to validate the impact of specific mutations on protein conformation and provide structural-level support for the underlying drug resistance mechanisms. Current diagnostic reliance on traditional culture and phenotypic drug sensitivity testing is often too slow to prevent the spread of MDR isolates or to guide timely, individualized treatment, with diagnostic delays often exceeding 2–3 weeks [25]. Therefore, whole-genome sequencing (WGS) is essential not only for tracing the lineage of local isolates but also for developing molecular tools that can bypass the delays of conventional testing [3, 17].

We therefore sequenced three clinical isolates (TI01, TI02 and TI03) collected between 2022 and 2024 from Fujian provinces. By integrating WGS, antifungal susceptibility testing (AFST) and functional annotation, we aimed to (i) map their phylogenetic position within the global population, (ii) systematically evaluate the impact of *CYP51B* locus amplification on the phenotypic landscape of *T. indotineae* to identify the genomic determinants of reduced azole susceptibility and (iii) use functional annotation to explore the core genome and virulence-related pathways that contribute to its pathogenicity. All custom scripts and bioinformatics pipelines used in this study have been deposited in the GitHub repository: https://github.com/microbial123/Trichophyton-indotineae.

## Methods

### Isolate collection and cultivation

In this study, three isolates of *T. indotineae* were all from the First Affiliated Hospital of Xiamen University and were isolated from the skin tissue of patients in 2022 (TI01) and 2023 (TI02 and TI03). The TI01 isolate was from a 35-year-old male patient, an engineer who had recently gone abroad. The TI02 isolate was from a 45-year-old female patient, a local resident. The TI03 isolate came from a 57-year-old female retiree, a local resident.

Septate fungal mycelium was detected by calcium fluorescence white reagent in the collected dander tissues. The dander tissues were inoculated with Sabouraud agar (Antu Biotechnology Company, Zhengzhou, China) and cultured at 27℃ for 1 week. Light yellow, ragged and powder-like colonies were observed on the medium. Under the microscope, a large number of single-celled spherical spores were distributed along the mycelia or arranged in grape-like clusters, and spiral mycelia were observed.

### Genomic DNA extraction and library preparation

Genomic DNA was extracted from fungal isolates using the Pathogen DNA Extraction Kit (model VT01, Hangzhou Di’an Biotechnology Co., Ltd., Hangzhou, China). To ensure efficient cell wall disruption of *T. indotineae,* samples underwent mechanical lysis via glass-bead homogenization (0.5 mm beads) prior to an automated magnetic-bead-based purification protocol.

Sequencing libraries were constructed using the Universal DNA Library Prep Kit (Hangzhou Di'an Biotechnology, China) with a standardized protocol. DNA was fragmented to an average insert size of 350 bp (range: 300–400 bp), confirmed via Bioanalyzer 2100 (Agilent). Following end-repair, adenylation and adapter ligation, library quality and concentration were quantified using a Qubit 4.0 Fluorometer (Thermo Fisher Scientific).

### Whole-genome sequencing

High-throughput sequencing was performed on the Salus Pro sequencing platform (Shenzhen Salus BioMed Co., Ltd. China) using a paired-end 150 bp (PE150) strategy. The sequencing reaction employed the Salus Universal Sequencing Reagent Kit based on the reversible-terminator sequencing technology. All procedures, including flow cell loading and cluster generation, were conducted following the standard Salus Pro operational protocols.

### Genome assembly and quality assessment

Raw Illumina data were first inspected for quality metrics with FastQC (v0.11.5, https://www.bioinformatics.babraham.ac.uk/projects/fastqc/) and then subjected to adapter removal and low-quality base trimming using fastp (v0.24.0) [26]. Clean reads were aligned to the *T. indotineae* reference genome (GCA_023065905.1) [27] using BWA-MEM (v0.7.18-r1243-dirty) [28]. Coverage depth across the *T. indotineae* genome was quantified with samtools depth (SAMtools v1.17) [29]. Optical and PCR duplicates were removed using samtools markdup-r, yielding deduplicated Binary Alignment Map (BAM) files for downstream variant discovery. SPAdes (v4.0.0) [30] was used to perform genome assembly from the sequencing data, yielding preliminary genome sequences. Subsequently, bcftools (v1.21; using htslib 1.21) [29] was employed to align the assembled contigs with the reference genome (GCA_023065905.1) and generate consensus sequences based on the detected variants, resulting in the final genome sequences. Finally, QUAST (v5.3.0) [31] was used to comprehensively evaluate the assembly quality, including metrics such as genome completeness, continuity and synteny with the reference genome.

Species identification was verified through alignment of the internal transcribed spacer (ITS) region with the reference sequence OR417031.1 and average nucleotide identity analysis was conducted using FastANI (v1.33) [32]. Genome completeness was evaluated with BUSCO (v5.2.2) [33] using the Fungi_odb12 lineage dataset.

### Phylogenetic reconstruction

To infer the evolutionary origin of the three *T. indotineae* isolates from mainland China, we queried the NCBI Sequence Read Archive on 20 November 2024 and retrieved WGS paired-end raw reads for 53 additional *T. indotineae* isolates collected worldwide, as well as the single-end raw reads for the reference genome (TIMM20114) (Table S1, available in the online Supplementary Material). These 53 genomes were processed with a different pipeline to the three newly sequenced isolates. Since the raw reads of the reference genome were generated using the PacBio third-generation sequencing platform, we performed quality control separately using fastplong (v0.4.1) [34] with the parameters ‘-m 20 -l 500 -A’, ultimately obtaining clean reads with an average quality higher than Q20 and a length ≥500 bp. Subsequently, pbmm2 (v26.1.99; commit v26.1.0-2-g60acde4) [35] was used to align the clean reads to the reference genome, generating a BAM alignment file. Yielding a final dataset of 57 high-quality genomes for phylogenomic analysis. Variant sites were called with BCFtools (v1.21, htslib 1.21) [29] using mpileup/call after excluding reads or bases with a Phred quality score less than 20. The resulting Variant Call Format (VCF) file was subsequently hard-filtered using the parameters ‘--minQ 40 --minDP 5 --max-missing 0.8’, corresponding to the following criteria: QUAL ≥40, missing ≤20%, depth ≥5 and indels were excluded. High-confidence SNPs were converted to phylip format with vcf2phylip.py (v2.9, https://github.com/edgardomortiz/vcf2phylip). A maximum-likelihood (ML) phylogeny was then inferred from the genome-wide SNPs of the 57 isolates. Phylogenetic inference was performed with IQ-TREE2 (v2.3.0) [36] under the best-fit nt substitution model selected by ModelFinder (-m MFP). The TVMe+G4 model was selected as the best-fit model according to the Bayesian Information Criterion. Branch support was assessed using 1,000 ultrafast bootstrap replicates (-B 1,000) and 1,000 SH-aLRT tests (-alrt 1,000). The final tree was visualized and annotated using Interactive Tree Of Life (iTOL v6).

### Detection of copy-number variations in candidate resistance-associated genes

To elucidate the genetic basis of the increasing minimum inhibitory concentration (MIC) trends among the three *T. indotineae* isolates, we performed genome-wide copy-number quantification to investigate whether dosage-dependent mechanisms – such as the expansion of resistance-associated genes – drive the incremental enhancement of antifungal resistance. By employing relative read-depth distribution benchmarked against the platform-matched isolate TI01 as a single-copy control, we eliminated discrepancies in G+C content and depth distribution inherent to the platform mismatch with the long-read reference genome (TIMM20114), ensuring a more robust evaluation of *CYP51B* expansion. Meanwhile, the conserved single-copy marker gene β-tubulin protein-coding gene (*TUB2*) was utilized as an internal control [37].

For genome-wide analysis, copy-number variations (CNVs) from three *T. indotineae* isolates were delineated using CNVkit (v0.9.11) [38]. Briefly, the genome was partitioned into contiguous 200 bp bins, followed by G+C-content correction to neutralize amplification bias. Isolate TI01, devoid of *CYP51B* amplification, served as the reference genome for copy-ratio estimation here. Per-bin read depths were calculated, normalized against the TI01 and segmented by circular binary segmentation to demarcate regions of continuous copy-number change, with a threshold of |log_2_ copy ratio|>1.5 used to define the occurrence of CNV. The cnvkit.py call command was executed with --ploidy 1 to convert segmented log_2_ ratios into discrete, integer copy-number states.

For locus-specific CNV analysis of the target *CYP51B* gene, we simultaneously profiled the single-copy marker *TUB2* for comparative reference to enhance the precision of CNV quantification. To precisely locate *CYP51B* and *TUB2*, their full-length coding sequences were aligned to the reference genome using BWA-MEM (v0.7.17-r1188), and only concordant mapping coordinates were retained. Base-level sequencing depths covering the full length and flanking regions of both *CYP51B* and *TUB2* were calculated via samtools. Depth normalization and correction were conducted using the average WGS depth as the baseline, and log_₂_-transformed values were generated to represent final copy number ratios. Line plot generated in R (v4.2.2) using ggplot2 (v3.5.2), providing a clear depiction of CNV across the analysed isolates. Employing the lm() function within R (v4.2.2) and utilizing the principle of least squares, linear regression analysis was performed on the *CYP51B* CNV and MIC data for 54 *T*. *indotineae* isolates to establish the final regression equation. Based on this equation, the MIC values for isolates TI01, TI02 and TI03 were further predicted and compared with their experimentally measured actual values.

### Genome-wide association analysis and sequence analysis

To investigate the association between TRB resistance phenotype and genome-wide variation, a genome-wide association study (GWAS) was performed on a collection of 56 isolates. SNP/InDel calling was performed with Snippy (v4.6.0, https://github.com/tseemann/snippy, default settings; minimum depth=20×, minimum base quality=30). The resulting VCFs were functionally annotated with SnpEff (v5.0e) [39] against the *T. indotineae* reference genome (GCA_023065905.1), generating high-impact variant predictions. TRB resistance was treated as a binary trait (resistant=1, non-resistant=0). Using the phylogeny_distance.py script in PySeer (v1.4.0) [40], a covariance matrix was generated from the phylogenetic tree of the 56 isolates to account for population structure. This matrix was incorporated as a random effect in the subsequent model to control for evolutionary relatedness (clonality) among isolates and reduce spurious associations. Thereafter, a logistic mixed-model was applied to examine whether the frequency distribution of each SNP differed significantly between the samples across the whole genome, and the corrected significance *P*-value (lrt-pvalue) was calculated for each SNP. Finally, a Manhattan plot was generated using the R package qqman (v0.1.9) to visualize the association between nonsynonymous SNPs (resulting in aa substitutions) and the resistance phenotype.

The *SQLE* gene sequences from TI01, TI02, TI03 and the reference genome were extracted separately using SAMtools (v1.22.1). Variant calling was performed with BCFtools (v1.21) to generate consensus sequences. Multiple sequence alignments were constructed using MAFFT (v7.525) [41], after which EMBOSS (v6.6.0) [42] translated the DNA alignments into aa sequence. Finally, the aligned protein sequences were rendered as publication-quality figures with Pymsaviz (v0.5.0).

### Protein modelling and docking analyses

To verify the hypothesis that the F397L mutation alters the drug-binding pocket structure of SQLE and induces TRB resistance, molecular docking was performed to predict the binding mode between TRB and wild-type/F397L-mutated SQLE using AutoDock Vina (v1.1.2). Prior to docking, a blastp (v 2.14.0) scan against the PDB database (updated to 2025-12-20) identified *Trichophyton rubrum* squalene monooxygenase (A0A289ZY54.1, 99.8% identity) as the template, and its full-length structure was retrieved from UniProt. The 3D structure of the small-molecule TRB was downloaded from the PubChem database (https://pubchem.ncbi.nlm.nih.gov/compound/Terbinafine). The ligand (PubChem CID 1549008) was energy-minimized using the MMFF94 force field (Tripos, SYBYL X-1.2). The protein–ligand complex was constructed using I-TASSER (with the best C-score/Z-score) and preprocessed via AutoDock Tools. The binding pose was then referenced to human SQLE-inhibitor structures (Protein Data Bank identifiers: 6C6N, 6C6P10) [17]. Before formal docking, protein preparation was conducted using PyMol (v3.1.1), including hydrogen addition and water molecule removal. A docking box was subsequently defined to enclose the active pocket of the protein. The small molecule and receptor protein in PDB format were converted to PDBQT format using PyMol (v3.1.1). Docking was then performed, and the conformation with the highest affinity score was selected as the optimal binding pose. Visualization analysis of this conformation was carried out using PyMol (v3.1.1) and Discovery Studio Visualizer (2021 Client).

To assess the structural conservation of SQLE across the *Trichophyton* genus, protein structures for *Trichophyton interdigitale* (A0A2R4H295), *Trichophyton mentagrophytes* (A0A289ZCP0) and *T. rubrum* (A0A289ZY54) were retrieved from the AlphaFold Protein Structure Database. The coordinate files (.pdb) were processed and visualized using PyMOL (v3.1.1).

### Gene structure and functional annotation

To establish a high-resolution genetic map and functional landscape of *T. indotineae*, we integrated gene structure prediction, orthology inference and multi-database functional profiling to define the core genetic architecture and pathogenic potential of the three clinical isolates. Genome masking was performed using the mask subcommand of Funannotate (v1.8.15) to identify and soft-mask repetitive sequences. The setup subcommand was then used to download the ascomycete-specific database. Finally, gene structure prediction was carried out with the predict subcommand, yielding a comprehensive set of predicted gene models. Align the aa sequences of protein-coding genes using OrthoFinder (v2.5.5) to identify orthologous genes among TI01, TI02, TI03 and the reference genome, and visualize the results using UpSet diagrams [43]. For pathogenicity analysis, we aligned predicted protein sequences and gene models against the Database of Fungal Virulence Factors (DFVF) using DIAMOND (v2.1.13.167), applying stringent filtering thresholds (E-value ≤1×10^-^⁵, bit-score ≥60) [44]. Functional annotation was performed using eggNOG-mapper to categorize the pangenome into Cluster of Orthologous Groups (COG), Gene Ontology (GO) and Kyoto Encyclopedia of Genes and Genomes (KEGG) categories. Subsequently, KEGG pathway analysis was conducted specifically on the subset of virulence-associated genes to elucidate the biological processes involved in the differentiated pathogenic potential between resistant and susceptible clinical isolates. Functional annotation was performed with eggNOG-mapper (v2.1.12) against eggNOG 6.0 database to retrieve orthologous groups and metabolic pathways. Hits were mapped to the pre-calculated eggNOG phylogeny to recover high-confidence orthologous groups; low-confidence matches were discarded. The filtered dataset provided robust functional annotations across COG, GO, KEGG, Carbohydrate-Active EnZymes (CAZy), Pfam and eggNOG taxonomic ortholog databases.

### *In vitro* antifungal susceptibility testing

AFST was determined using the broth microdilution method following Clinical and Laboratory Standards Institute (CLSI M38-A3) guidelines. Following clinical isolation and purification, isolates were adjusted to a final inoculum of 1×10^3^ to 3×10^3^ c.f.u. ml^−1^ in RPMI 1640 medium. The fungal growth in each well was quantified via spectrophotometry using a Multiskan SkyHigh Microplate Reader (Thermo Fisher Scientific, USA) at a wavelength of 530 nm. The MIC was defined as the lowest concentration resulting in a ≥80% reduction in OD compared to the drug-free growth control. Due to the absence of official CLSI or European Committee on Antimicrobial Susceptibility Testing (EUCAST) clinical breakpoints for *T. indotineae,* AFST results were interpreted based on previously established epidemiological cutoff values (ECVs). For TRB, MIC >0.2 g ml^−1^ was utilized as the threshold for resistance, as this value has been consistently associated with *SQLE* mutations and clinical recalcitrance in recent multicentre studies. Specifically, the following ECVs were applied in this study: for TRB, MIC >0.2 mg l^−1^; for fluconazole (FLC), MIC >32 mg l^−1^; for ITC, MIC >0.5 mg l^−1^; for VRC, MIC >2 mg l^−1^; and for posaconazole (POS), MIC >0.5 mg l^−1^ [7]. In order to ensure statistical robustness, all assays were performed in triplicate across independent experiments. Standard quality control was maintained using *Candida krusei* (ATCC 6258) and *Candida parapsilosis* (ATCC 22019). Plates were incubated at 30 °C, and OD readings were recorded on days 5 and 7, with the day 7 stable-growth data utilized for subsequent correlative modelling.

## Results

### Clinical manifestations and fungal morphological characteristics

To characterize the clinical presentation and fungal morphology of the *T. indotineae* infection, we documented the patient’s dermatological manifestations and conducted macroscopic and microscopic examinations of isolate TI03. A 57-year-old retired woman presented with well-demarcated, erythematous plaques covered by fine scales on the posterior thighs and gluteal region, accompanied by intense pruritus and intermittent burning or stinging sensations, consistent with the clinical manifestation of dermatophytosis (Fig. S1a). Isolate TI03 exhibited macro- and microscopic features consistent with the genus *Fusarium*. On PDA, colonies were flat, white and displayed a suede-like surface (Fig. S1b). Microscopically, abundant cigar- to club-shaped macroconidia (20–50×6–8 µm, 3–4 septa, smooth- and thin-walled, narrow basal attachment) dominated, accompanied by numerous subspherical to pyriform microconidia and occasional spiral hyphae (Fig. S1c).

### The genomic characteristics of isolates TI01, TI02 and TI03

We established the genomic landscape of the three clinical *T. indotineae* isolates by leveraging high-throughput WGS and comprehensive genomic assembly. Sequencing generated a total of 234 M raw reads (mean±sd: 234.38±49.14 M), which after quality filtering yielded 234 M (mean±sd: 234.32±49.13 M) clean reads, providing an average coverage of over 1,300×. The target sequencing depth averaged 287× for *SQLE* and 1,752× for *CYP51B*.

To reveal the genomic characteristics of three isolates of *T. indotineae*, we conducted WGS and presented a summary of the isolates’ genomic features in Table 1. The assembled genomes of the three isolates were uniformly ~22.26 Mb in length and harboured 7,316–7,401 predicted protein-coding genes, which correspond to ~57% of each genome. G+C content was consistently 49 mol% across all isolates.

**Table 1.** Genome assembly information of isolates TI01, TI02 and TI03

Feature	Value		
	TI01	TI02	TI03
Total length (bp)	22,257,530	22,260,331	22,254,680
Protein coding genes	7,401	7,369	7,316
Gene length (bp)	12,779,062	12,653,609	12,676,063
Gene average length (bp)	1726.67	1717.14	1732.65
Gene/genome (%)	57.41	56.84	56.96
G+C content (%)	48.73	48.73	48.73
N50 (bp)	8,009,480	8,011,058	8,008,634
N90 (bp)	4,065,257	4,065,661	4,064,632
Contig number	3	3	3
Complete BUSCOs (%)	98.8	98.8	98.8
Depth	1,962×	1,395×	1,382×

### Clade assignment of three clinical *T. indotineae* isolates relative to global reference isolates

**Fig. 1.**
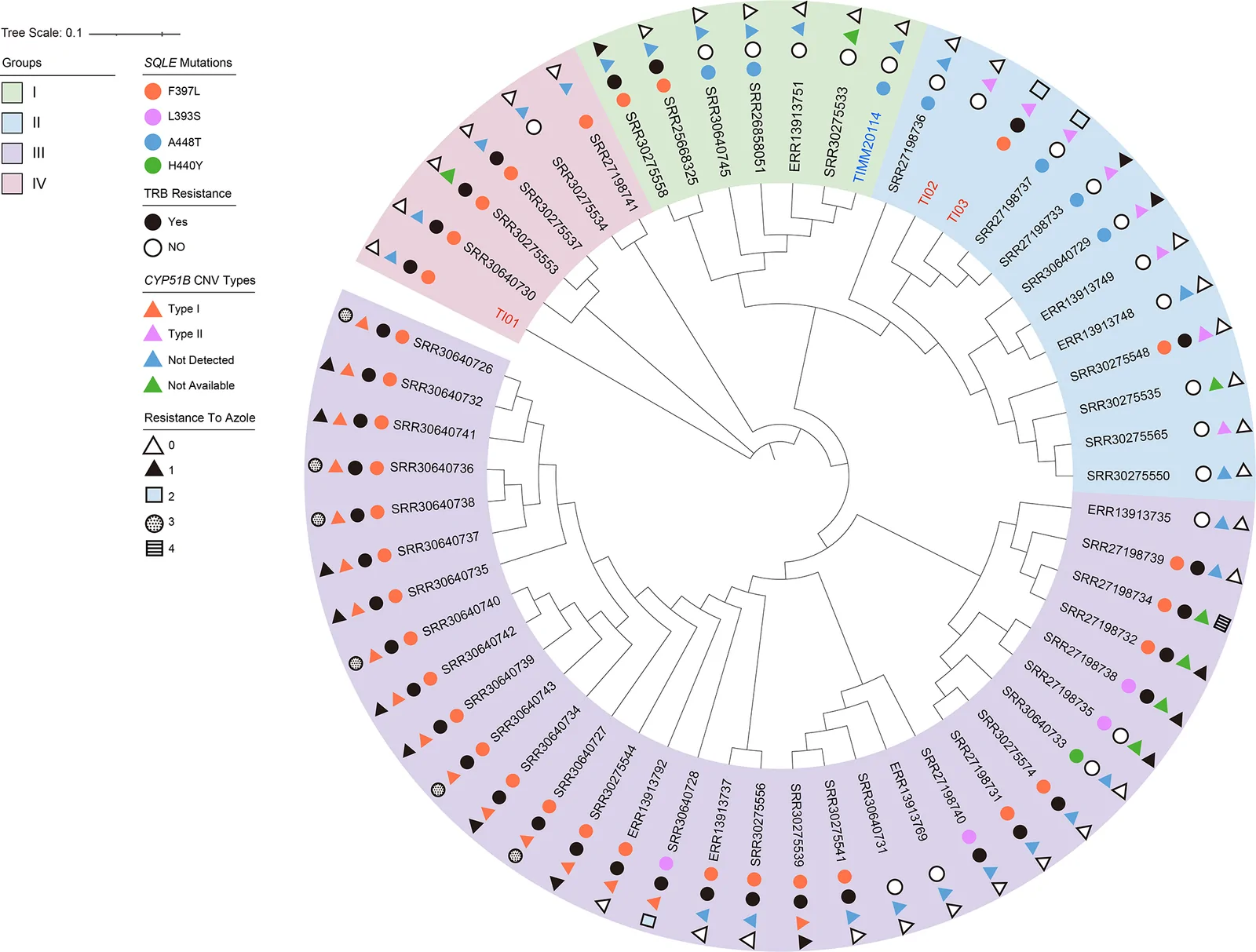
Phylogenomic analysis and drug resistance profiling of 57 global *T. indotineae* isolates. The ML tree was reconstructed based on genome-wide SNPs derived from WGS data, rather than ITS sequences, using the TVMe+G4 substitution model. Branch support was evaluated via 1,000 ultrafast bootstrap (UFBoot) replicates and SH-aLRT tests (values indicated at nodes). Genome sequences for isolates TI01–TI03 (generated in this study) are highlighted in red; the reference strain TIMM20114 is highlighted in blue. Genome sequences and matched MIC values for the remaining 53 isolates were retrieved from NCBI and the original publications, respectively. Note: Background shading delineates clades I–IV. Concentric rings (inner to outer) represent: (1) *SQLE* mutations, designated by coloured circles; (2) TRB resistance, with black and white circles indicating resistant and susceptible isolates, respectively; (3) *CYP51B* CNVs, with coloured triangles indicating CNV types (not detected, single copy; not available, CNV type could not be determined); and (4) multidrug azole resistance, with distinct geometric shapes and colours indicating the number of azoles (0–4) among FLC, ITC, VRC and POS to which resistance was observed. The scale bar represents substitutions per site.

To determine the phylogenetic position of our clinical isolates within the global *T. indotineae* population, we constructed an ML phylogenomic tree incorporating publicly available isolates collected globally. Fig. 1 presents the results of the evolutionary tree constructed by 57 isolates. TI01 belongs to clade IV, while TI02 and TI03 both belong to clade II. Table S1 compiles susceptibility and resistance-genotype data for 57 global *T. indotineae* isolates. Among these 57 isolates, 33 isolates (including TI01 and TI03) harboured the *SQLE* Phe397Leu mutation, 6 isolates carried the A448T mutation and 4 isolates exhibited the L393S mutation. Of the isolates with the F397L mutation, AFST revealed TRB resistance in 32 isolates (one unavailable), including TI01 and TI03, demonstrating a strong correlation between the F397L mutation and the TRB-resistant phenotype. The *CYP51B* CNV type of each isolate and its AFST results for the four azoles (FLC, ITC, VRC and POS) are also detailed in Table S1.

### Association between *CYP51B* copy-number variation and azole drug resistance

We conducted a genome-wide CNV analysis focusing on the *CYP51B* gene locus to investigate the genomic mechanisms underlying azole resistance. This section presents the relationship between *CYP51B* CNV and azole MICs across clinical isolates.

Comparative genomic analysis revealed a striking expansion of the *CYP51B* gene (located at JAJVHL010000003.1 : 2,121,925–2,123,699) in azole-resistant isolates relative to the single-copy baseline established by the reference genome TIMM20114. As illustrated in the genome-wide CNV landscape (Fig. S2), *CYP51B* exhibited the highest amplitude of copy-number gain across the entire *T. indotineae* genome, corresponding to the region highlighted by the blue line in the figure.

**Fig. 2.**
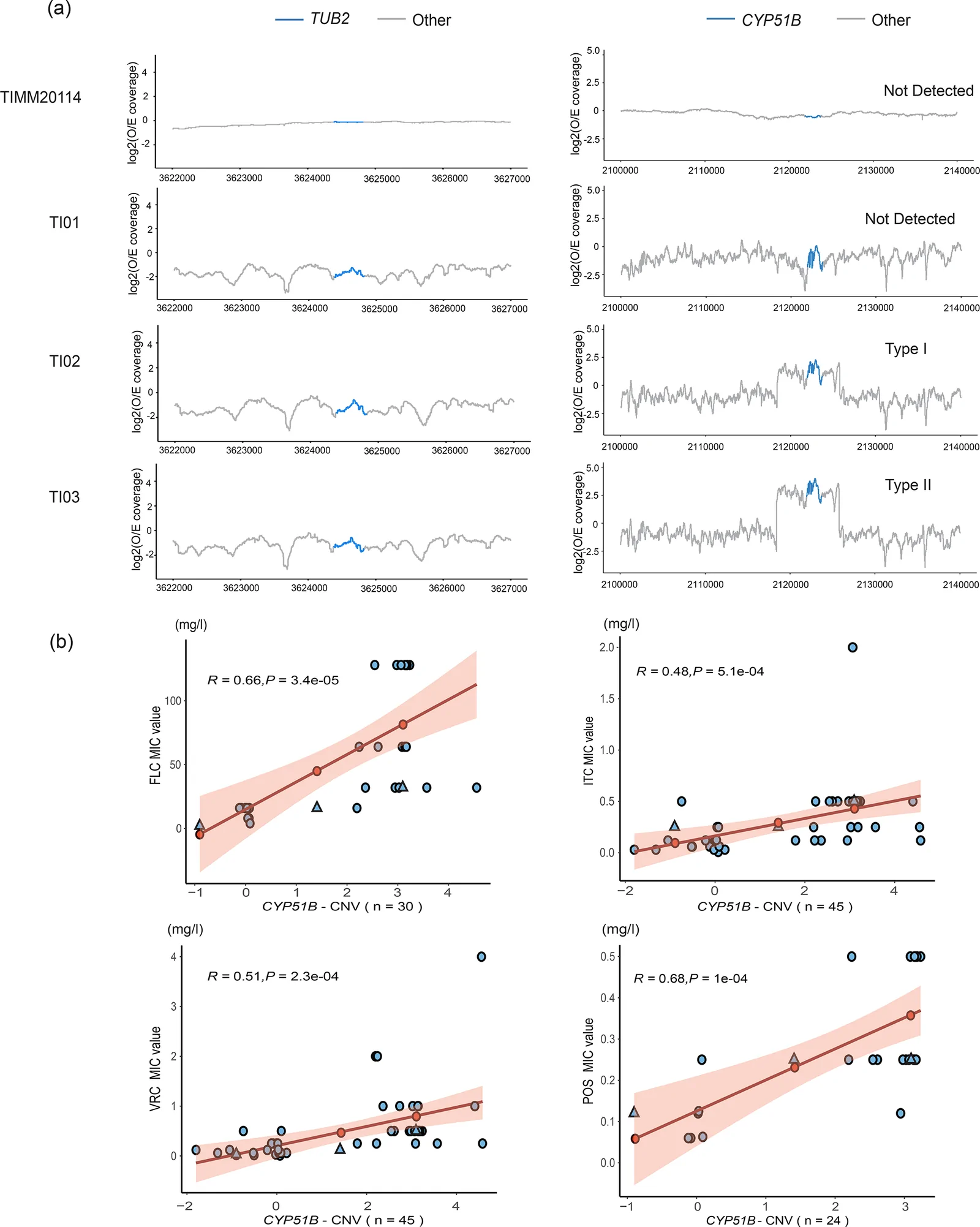
Characterization of *CYP51B* CNVs and their correlation with azole resistance in *T. indotineae*. (**a**) High-resolution CNV mapping of the *TUB2* and *CYP51B* genes. Visualization of copy number distribution within the genomic regions JAJVHL010000001.1 : 3,600,000–3,630,000 and JAJVHL010000003.1 : 2,100,000–2,140,000 (X-axis). The *TUB2* (3,624,382–3,624,823, left) and *CYP51B* gene regions (2,121,925–2,123,699, right) are highlighted with blue dots. The Y-axis represents the Observed/Expected (O/E) ratio per bp. (**b**) Correlation between *CYP51B* CNV and azole MICs. Linear regression analysis between *CYP51B* CNV values (X-axis) and MICs (Y-axis) for FLC (*n*=30), ITC (*n*=45), VRC (*n*=45) and POS (*n*=24). Blue circles denote the experimental data of the public isolates used for model construction. For isolates TI01, TI02 and TI03, predicted MIC values (based on CNV data) and their corresponding experimentally determined MICs are indicated by red dots and blue triangles, respectively. Pearson correlation coefficients R and *P*-values are provided for each drug; *P*<0.05 was considered statistically significant. Note: CNV represents log_2_-normalized copy ratios. Data are presented as log_2_ fold changes in the *CYP51B* copy ratio relative to the TI01 genome. A log_2_ value of 0 represents a baseline single-copy status (identical to TI01), while positive values (log_2_>0) indicate gene expansion; for example, a log_2_ value of 1.0 corresponds to approximately a twofold increase in copy number. Negative values indicate a copy number lower than the genome-wide average, signifying a genomic loss.

Detailed analysis of specific isolates revealed distinct genomic profiles. Fig. 2a delineates the landscape of CNVs surrounding the *TUB2* (JAJVHL010000001.1 : 3,600,000–3,630,000) and *CYP51B* locus (JAJVHL010000003.1 : 2,100,000–2,140,000). While isolate TI01 exhibited baseline copy number levels at the *CYP51B* locus (CNV range: −2.19 to 0.1), prominent genomic amplifications of this gene were detected in TI02 (CNV range: 0.03–2.29) and TI03 (CNV range: 1.78–4.05). TI03 displayed a higher log_2_ copy ratio (Y-axis) for *CYP51B* compared with TI02, demonstrating more substantial copy number expansion of this target gene. These elevated CNV values at the *CYP51B* locus suggest a direct correlation between increased gene dosage and the observed antifungal MIC values.

To further quantify this relationship, we established linear regression models correlating the MICs with the *CYP51B*-CNV. As illustrated in Fig. 2b, a significant positive correlation was observed between the *CYP51B* CNV and the MIC values for four clinical azoles (FLC, VRC, ITC and POS) across the tested isolates. All four azoles exhibited statistically significant positive correlations, with strong correlations observed for FLC (*R*=0.66, *P*=3.42×10^−5^, *n*=30) and POS (*R*=0.68, *P*=1.03×10^−4^, *n*=24). Relatively strong correlations were maintained for VRC (*R*=0.51, *P*=2.32×10^−4^, *n*=45) and ITC (*R*=0.48, *P*=5.1×10^−4^, *n*=45). For detailed results, see Table S2. Notably, the predicted MICs for representative isolates TI01, TI02 and TI03, derived from these linear models, closely approximated the experimentally determined values.

### Genomic association and molecular docking analysis of *SQLE* mutations in *T. indotineae*

To explore genetic determinants of TRB resistance and elucidate the structural impact of *SQLE* mutations, we performed GWAS and molecular docking simulations. This section reports associations between *SQLE* mutations and TRB resistance, along with structural analyses of drug–target interactions.

**Fig. 3.**
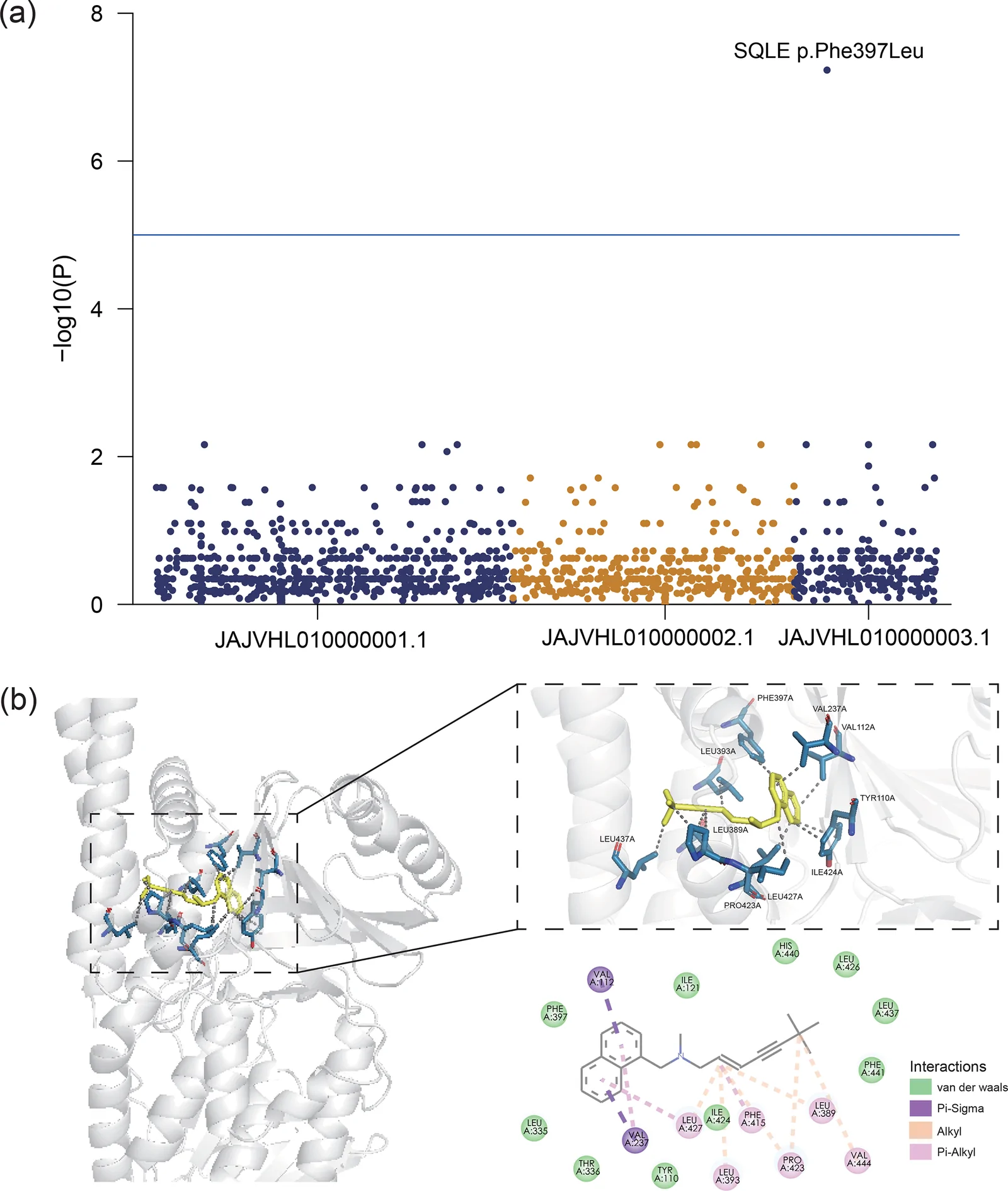
*SQLE* gene mutations in *T. indotineae* and *in silico* docking analyses. (**a**) The Manhattan plot of GWAS results showing the association between SNPs and drug resistance phenotypes at the genome-wide scale. The ‘−log_10_(*P*)’ is significant *P* value after correction. The horizontal blue line indicates the genome-wide significance threshold (*P*=1×10^−5^). (**b**) Global view of the SQLE binding pocket. The ribbon-transparent structure represents the *SQLE* homology model. TRB (represented as yellow sticks) is docked within the hydrophobic cavity composed of key residues including Phe397 and Leu393 (depicted as blue sticks). Upper right inset: A magnified view illustrates the specific interactions between the naphthalene moiety of TRB and the primary hydrophobic residues; grey dashed lines denote hydrophobic interactions, with bond distances indicated in Ångströms (Å). Lower right inset: A two-dimensional interaction diagram of the SQLE–TRB complex. Colour coding for the interaction types is as follows: green represents van der Waals forces, purple denotes Pi-Sigma, orange indicates alkyl interactions, and pink signifies pi-alkyl interactions.

Genome-wide scanning identified the SQLE p.Phe397Leu mutation as the most significant genomic determinant of TRB resistance (*P*=5.88 E-08), exhibiting a stronger association than any other locus across the entire genome (Fig. 3a). Fig. S3a illustrates the alignment of SQLE protein sequences between isolates TI01, TI02, TI03 and the reference sequence. The F397L mutation in the *SQLE* gene was consistently detected in isolates TI01 and TI03. Conversely, no aa substitutions were observed in the *SQLE* sequence of isolate TI02, which remained identical to the wild-type reference.

Among the five constructed models, SQLE_Model_1 (Fig. S3b) exhibited an experimental TM-score (exp. TM-score) of 0.94±0.05 and an experimental root mean square deviation (exp. RMSD) of 3.9±2.7 Å. This model achieved the optimal C-score (1.6) and was therefore selected for molecular docking analyses. Ramachandran plot analysis revealed that 93.7% of the residues were located in the most favoured regions, while 6.3% were in the additionally allowed regions (Fig. S3c). The overall quality factor of SQLE evaluated by the ERRAT program was 94.2 (Fig. S3d). The binding interface between TRB and SQLE is primarily stabilized by a robust network of hydrophobic interactions. Structural analysis reveals that the naphthalene moiety of TRB is accommodated within a specialized hydrophobic pocket defined by Phe397 and Leu393 (Fig. 3b). Computational modelling identified 14 distinct hydrophobic contact sites involving 10 key aa residues: Tyr110, Val112, Ile121, Val237, Leu389, Leu393, Phe397, Phe415, Pro423, Leu427 and Leu437.

Within this pocket, Phe397 serves as the critical anchoring residue, establishing two primary hydrophobic contacts with exceptionally short distances of 2.74 and 3.10 Å. Specifically, the naphthalene ring of TRB engages in hydrophobic stacking with the aromatic side chain of Phe397. Concurrently, the allylamine side chain of the ligand interacts with the alkyl side chain of Leu393 through a combination of steric complementarity and hydrophobic effects. These interactions are consistent with recent genomic and structural characterizations of *T. indotineae*, which underscore the significance of these residues in mediating drug susceptibility [17]. Notably, homology modelling indicates that the Phe397Leu substitution – a common resistance mutation – leads to a collapse of the predicted hydrophobic pocket, significantly hindering TRB binding affinity.

### Functional annotation analysis of genes of isolates TI01, TI02 and TI03

**Fig. 4.**
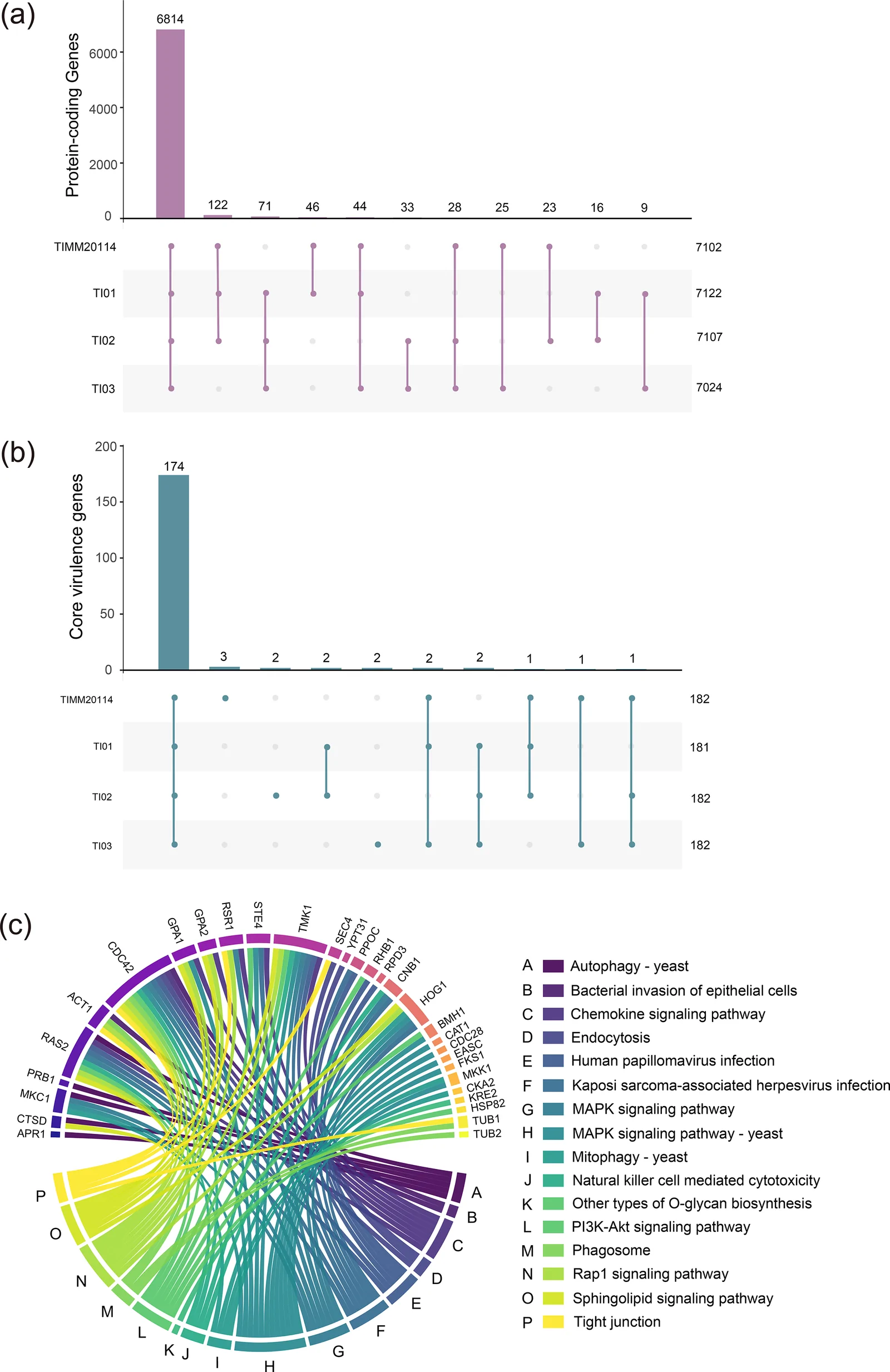
Comparative genomic analysis of core protein-coding and virulence-associated genes in *T. indotineae*. (**a**) Intersection of protein-coding genes. Shared and unique protein-coding genes among three Chinese *T. indotineae* isolates and the Indian reference isolate (TIMM20114). (**b**) Distribution of core virulence genes. Comparison of virulence-associated genes identified via the DFVF across the four analysed genomes. (**c**) KEGG pathway classification of identified virulence genes. Functional mapping of the predicted virulence genes to their respective KEGG pathways, representing the biological processes associated with fungal pathogenicity. Note: UpSet plots in panels (**a**) and (**b**) illustrate the intersection relationships among the four *T. indotineae* isolates. In these plots, the matrix of connected dots indicates specific intersection sets; the upper bar chart represents the number of genes within each intersection set; and the horizontal bars denote the total number of annotated genes per isolate.

Aiming to characterize the functional genomic landscape and virulence potential of the three *T. indotineae* isolates, we conducted a comparative genomic analysis and virulence gene annotation. This cross-strain comparison revealed 6,814 genes shared across all four isolates, with 170, 138, 85 and 205 isolate-specific genes unique to TI01, TI02, TI03 and the reference genome (GCA_023065905.1), respectively (Fig. 4a). Virulence annotation against the DFVF database identified 174 core virulence genes that were shared among the three Chinese isolates (Fig. 4b). Additionally, when virulence annotation was performed using both the DFVF and PHI-BASE databases, 25 core virulence genes were found to be common to the three isolates. Among the annotated virulence genes from these two databases, 149 genes were exclusive to the DFVF database, while 453 genes were unique to the PHI-BASE database (Fig. S4a).

Pathways containing more than five virulence genes are shown in Fig. S4b. Among these pathways, mitogen-activated protein kinase 1 (*TMK1*), Ras family GTPase 2 (*RAS2*), cell division control protein 42 (*CDC42*), high osmolarity glycerol 1 (*HOG1*) and guanine nucleotide-binding protein alpha subunits 1 and 2 (*GPA1/2*) were identified across 24, 22, 18, 15 and 15 distinct pathways, respectively. Fig. 4c illustrates the KEGG pathways of virulence genes associated with fungal infection and host–pathogen interactions. The MAPK signalling harboured the largest gene set, including *CDC42*, the 14-3-3 protein homologue *BMH1*, steile 4 (*STE4*), early ascus-specific protein *EASC*, *GPA1*, *HOG1*, mitogen-activated protein kinase kinase 1 (*MKK1*), catalase 1 (*CAT1*), TMK1, 1,3-β-glucan synthase component *FKS1*, cell wall integrity kinase *MKC1* and cell division control protein 28 (*CDC28*). *O*-glycan synthesis was represented by the α-1,2-mannosyltransferase *KRE2,* while genes associated with endocytosis, specifically *CDC42*, the Rab family GTPase *YPT31*, and secretory protein *SEC4*, were prominent within the virulence-related functional landscape.

### Comparison of the antifungal susceptibility test

In order to comprehensively assess the antifungal resistance profiles of the three clinical *T. indotineae* isolates, AFST was performed against nine antifungal agents. TI01 exhibited the lowest azole MICs (ITC 0.25 mg l^−1^, VRC and MCZ 0.03 mg l^−1^) and the highest TRB MIC (4 mg l^−1^). TI02 showed eightfold elevation in FLC MIC (16 mg l^−1^) compared with TI01, whereas it was extremely susceptible to TRB with an MIC of only 0.015 mg l^−1^. In contrast, TI03 showed the highest MICs across all azoles – ITC 0.5 mg l^−1^, FLC 32 mg l^−1^, MCZ 1 mg l^−1^, VRC 0.5 mg l^−1^ – and for TRB (8 mg l^−1^). These data are detailed in Table 2.

**Table 2.** MIC (mg l^−1^) of *T. indotineae* species against nine drugs

Isolate	ITC	FLC	MCZ	VRC	KTZ	POS	ISA	TRB	AMB
**TI01**	0.25	2	0.03	0.03	0.125	0.12	0.12	4	0.5
**TI02**	0.25	16	0.12	0.12	0.125	0.25	0.5	0.015	1
**TI03**	0.5	32	1	0.5	0.5	0.25	1	8	1

AMB, Amphotericin B; ISA, Isavuconazole; KTZ, Ketoconazole; MCZ, Miconazole.

## Discussion

By integrating WGS, AFST and structural functional annotation, this study deciphers the genomic landscape of *T. indotineae* in China. Building upon prior research that identified *CYP51B* expansion as a categorical marker of resistance [7, 23, 27], our work advances this understanding by exploring a preliminary quantitative model that correlates genomic CNV with MIC values. Due to the absence of official CLSI or EUCAST clinical breakpoints for *T. indotineae*, this study seeks to bridge the gap between genomic profiling and clinical MIC prediction, establishing a valuable framework for the transition from qualitative observation to quantitative, genome-based susceptibility forecasting.

Phylogenomic analysis assigned our three Fujian isolates to two distinct clades (Groups II and IV), distinguishing them from the Group III cluster previously reported in eastern China (Jiangsu) [7]. Although the current sample size is limited to three newly sequenced strains, the identification of multiple clades within a single region tentatively suggests a degree of genetic diversity among *T. indotineae* in China [45, 46].

Employing an integrated landscape genomic and structural approach, we demonstrate that *T. indotineae* deploys a dual-track resistance mechanism: *CYP51B* amplification confers a quantitative advantage against azole antifungals, whereas the SQLE p.Phe397Leu mutation alters the structural configuration of the drug-binding pocket, leading to a significant reduction in the binding affinity of TRB – thus establishing a robust framework for genome-guided clinical interventions. Distinct from ancillary substitutions (e.g. L393F/S, F415Y and H440Y) that yield incremental susceptibility shifts [17, 47], the SQLE p.Phe397Leu mutation acts as a decisive ‘on–off’ switch for high-level TRB resistance. This observation aligns with Caplan *et al*. [17] and is further corroborated by Rhodes *et al*. [48], whose multinational treeWAS analysis (*P*<0.0001) identified F397L as the primary driver of MIC elevations ranging from 0.5 to ≥16 mg l^−1^. The SQLE protein architecture is highly conserved across the *Trichophyton* genus, with the catalytic domain of *T. indotineae* aligning closely with those of *T. interdigitale*, *T. mentagrophytes* and *T. rubrum* (Fig. S3e). This structural homology, consistent with recent genetic insights into the *T. mentagrophytes* complex [49]. In *T. indotineae*, the F397L substitution appears to induce a localized structural distortion and pocket collapse within the SQLE hydrophobic tunnel, resulting in a structural incompatibility that prevented the identification of stable TRB docking orientations. This architectural shift represents a primary evolutionary strategy for resistance, one that is highly consistent with the SQLE binding pocket model described by Caplan *et al*. [17]. Although Rhodes *et al*. noted rare F397L occurrences in susceptible isolates [48], our findings of perfect genotype–phenotype concordance support its general utility as a highly predictive TRB-resistance marker. Nonetheless, the low-level heterogeneity reported in larger cohorts highlights the need to couple genotypic screening with phenotypic testing to prevent false positives. Definitively decoupling this causal relationship – and uncovering potential epistatic modifiers behind global phenotypic complexities – will require further experimental validation, such as site-directed mutagenesis.

The elucidation of the relationship between *CYP51B* CNV and azole resistance represents a central highlight of this study. Notably, a progressive increase in antifungal resistance was observed from TI01 to TI03. TI01 exhibited low MIC values to all tested azoles (FLC: 2 mg l^−1^, ITC: 0.25 mg l^−1^, VRC: 0.03 mg l^−1^, POS: 0.12 mg l^−1^). In contrast, TI02 showed markedly elevated FLC resistance (FLC: 16 mg l^−1^), and TI03 further developed the highest resistance level (FLC: 32 mg l^−1^) with concomitant increases in ITC and VRC MICs. This stepwise elevation in azole resistance coincided with a gradual increase *CYP51B* copy number (log_2_ copy ratio from −0.90 to 3.10), with *TUB2* serving as an internal control for copy number quantification. This observation suggests that amplification of *CYP51B* may drive the progressive MDR phenotype across isolates TI01–TI03. The emergence of *T. indotineae* marks a significant paradigm shift towards target overexpression via complex structural variations. While previous studies identified *CYP51B* expansion as a general marker [7, 23, 27], we established, for the first time, a linear regression model between CNV and MIC values, showing a high degree of predictive accuracy for isolates TI01–TI03 (*P*<0.05). Compared to the long turnaround time of traditional phenotypic tests, this genomic-based susceptibility prediction offers a rapid, reliable molecular reference, facilitating the transition towards precision genomic medicine for managing recalcitrant dermatophytosis. The role of *CYP51B* in azole resistance is well-documented within the *Trichophyton* genus, where resistance typically stems from point mutations in species like *T. rubrum* and *T. mentagrophytes* [49]. The amplification of *CYP51B* observed in *T. indotineae* echoes the resistance mechanism of its ortholog, *ERG11*, reported in *Candida* species [50], suggesting a conserved evolutionary strategy across diverse fungal taxa to overcome azole pressure through target gene duplication. While our results highlight the correlation between *CYP51B* CNV and MIC values, it is important to note that mRNA overexpression can also be driven by other mechanisms. These include mutations within promoter regions or alterations in transcriptional regulatory networks, as observed in other fungal models [51, 52].

Our comparative genomic analysis reveals a relatively conserved genetic architecture among the three *T. indotineae* isolates sequenced in this study. The identification of 6,814 core genes and a shared repertoire of 174 virulence factors suggests a potentially common pathogenic baseline specific to these particular isolates. This local observation tentatively aligns with the remarkably low nt diversity (mean ~147 SNPs) previously reported across a global collection of 103 isolates [48]. However, given the limited and early subset of global genomes available to date, this observed uniformity should be interpreted with caution and treated as a provisional insight; whether *T. indotineae* truly maintains strict genetic homogeneity across disparate geographic populations remains to be validated using larger, more comprehensive genomic cohorts. Consequently, the 174 core virulence genes annotated via DFVF are best characterized at this stage as putative pathogenicity determinants that warrant further experimental and functional validation [53]. Specifically, the MAPK signalling pathway was identified as a key regulatory hub, containing 12 critical virulence genes (e.g. *CDC42*, *HOG1*) that govern stress adaptation and host invasion [54]. *CDC42* is a well-established master regulator of fungal morphogenesis and polar growth facilitating the robust hyphal development and tissue invasion [55]. Furthermore, pathways such as endocytosis and KRE2-mediated *O*-glycan biosynthesis likely support host colonization and immune evasion [56]. During infection, these factors coordinate the core stages of adhesion, germination and invasion [57], where fungal-induced damage and the subsequent host inflammatory response collectively drive disease progression [58].

Despite these significant findings, several limitations of this study should be acknowledged. First, while this study incorporates an extensive integration of publicly available genomic datasets, the current sampling of clinical isolates from mainland China represents a preliminary cohort. While these data provide valuable initial insights, a larger and more geographically diverse sample set would further enhance the granularity of our inferences regarding the transmission pathways and evolutionary trajectories of *T. indotineae* within the region. Second, our CNV quantification was based on a read-depth distribution strategy benchmarked against a platform-matched, single-copy isolate. While this approach offers a preliminary estimation and helps mitigate platform-specific biases, bioinformatic tools can be inherently conservative when resolving extremely high-expansion events. We suggest that future investigations incorporate reverse-transcription polymerase chain reaction (RT-PCR) as a parallel analytical standard to more precisely confirm CNV types. Furthermore, integrated multi-omics research, encompassing both genomic and transcriptomic datasets, will be essential to further elucidate the molecular interplay between *CYP51B* gene dosage and azole resistance.

## Supplementary material

10.1099/mgen.0.001812 Supplementary Material 1.

10.1099/mgen.0.001812 Supplementary Material 2.

## Abbreviations

AFST: antifungal susceptibility testing
AMB: amphotericin B
BAM: Binary Alignment Map
CAT1: catalase 1
CAZy: Carbohydrate-Active EnZymes
CDC28: cell division control protein 28
CDC42: cell division control protein 42
CLSI: Clinical and Laboratory Standards Institute
CNV: copy-number variation
COG: Cluster of Orthologous Groups
*CYP51B*: cytochrome P450 family 51 subfamily B member 1
DFVFs: Database of Fungal Virulence Factor
ECV: epidemiological cutoff value
EUCAST: European Committee on Antimicrobial Susceptibility Testing
FLC: fluconazole
GO: Gene Ontology
GPA1/2: guanine nucleotide-binding protein alpha subunits 1 and 2
GWAS: genome-wide association study
HOG1: high osmolarity glycerol 1
ISA: isavuconazole
ITC: itraconazole
ITS: internal transcribed spacer
KEGG: Kyoto encyclopedia of genes and genomes
KTZ: ketoconazole
MCZ: miconazole
MDR: multidrug resistance
MIC: minimum inhibitory concentration
MKK1: mitogen-activated protein kinase kinase 1
ML: maximum-likelihood
PHI-BASE: pathogen host interactions database
POS: posaconazole
RAS2: Ras family GTPase 2
RT-PCR: reverse-transcription polymerase chain reaction
SQLE: squalene epoxidase
STE4: steile 4
TMK1: mitogen-activated protein kinase 1
TRB: terbinafine
TUB2: β-tubulin protein-coding gene
VCF: Variant Call Format
VRC: voriconazole
WGS: whole-genome sequencing
