# Supplementary material for: Deciphering the genomic landscape of Trichophyton indotineae in China and exploring a correlative model for azole resistance prediction
Source: Microb Genom. 2026 Sep 16;12(9):001812. doi: 10.1099/mgen.0.001812 (PMC13580827; doi:10.1099/mgen.0.001812)
Supplement: Supplementary file 1 [file mgen-12-01812-s001.pdf]

Figure S1

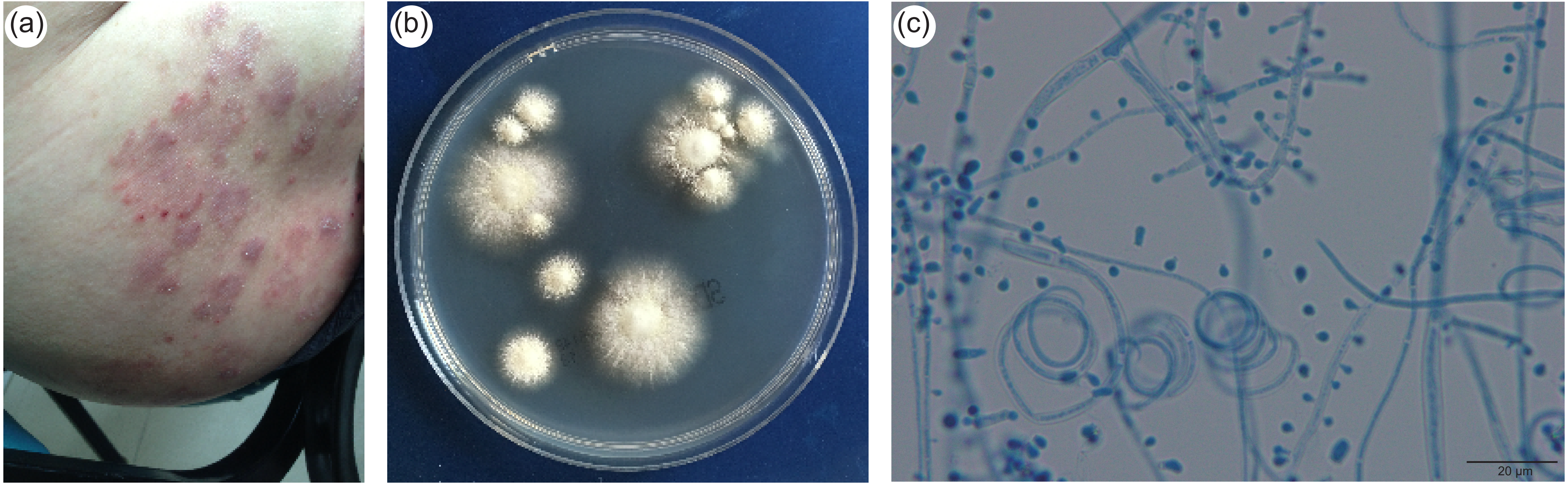

Figure S2

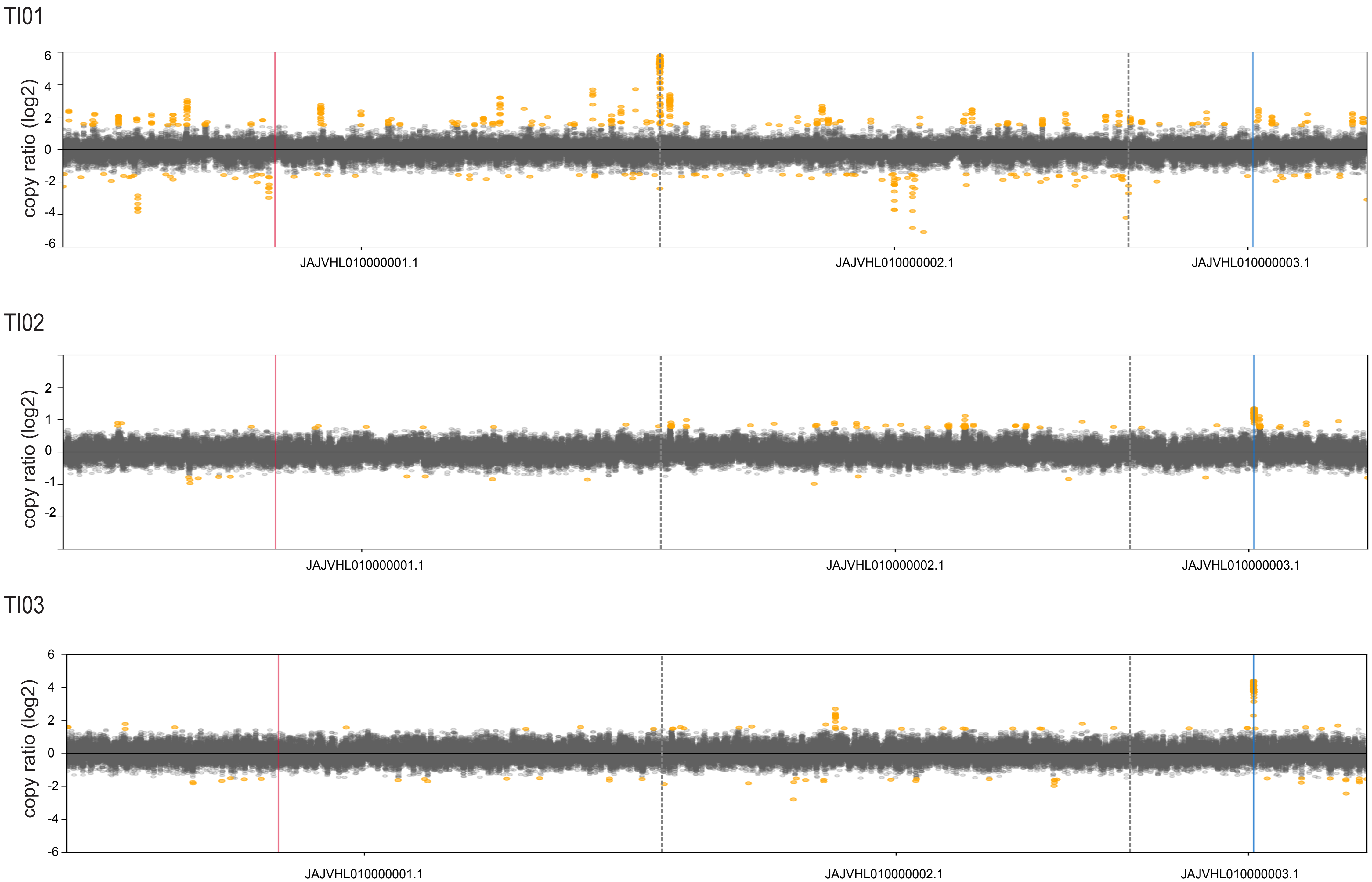
TI01
copy ratio (log2)
6
4
2
0
-2
-4
-6
JAJVHL010000001.1
JAJVHL010000002.1
JAJVHL010000003.1
TI02
copy ratio (log2)
2
1
0
-1
-2
JAJVHL010000001.1
JAJVHL010000002.1
JAJVHL010000003.1
TI03
copy ratio (log2)
6
4
2
0
-2
-4
-6
JAJVHL010000001.1
JAJVHL010000002.1
JAJVHL010000003.1

Figure S3

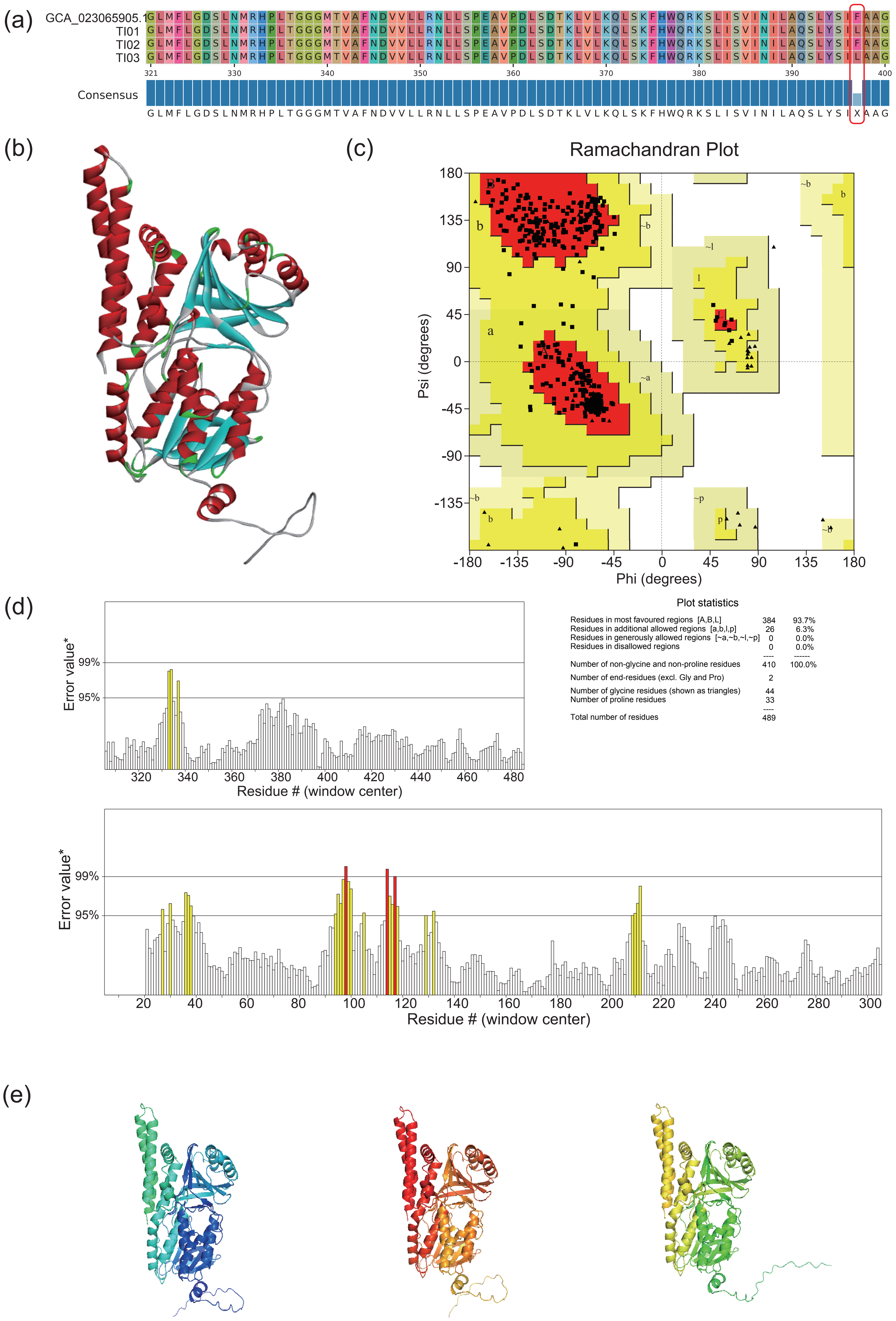

(a)
GCA_023065905.1 GLMFLGDSLNMRHPLTGGGMTVAFNDVVLLRNLLSPEAVPDLSDTKLVLKQLSKFHWQRKSLISVINILAQSLYSIFAAG
TI01 GLMFLGDSLNMRHPLTGGGMTVAFNDVVLLRNLLSPEAVPDLSDTKLVLKQLSKFHWQRKSLISVINILAQSLYSILAAG
TI02 GLMFLGDSLNMRHPLTGGGMTVAFNDVVLLRNLLSPEAVPDLSDTKLVLKQLSKFHWQRKSLISVINILAQSLYSIFAAG
TI03 GLMFLGDSLNMRHPLTGGGMTVAFNDVVLLRNLLSPEAVPDLSDTKLVLKQLSKFHWQRKSLISVINILAQSLYSILAAG
321 330 340 350 360 370 380 390 400
Consensus
G L M F L G D S L N M R H P L T G G G M T V A F N D V V L L R N L L S P E A V P D L S D T K L V L K Q L S K F H W Q R K S L I S V I N I L A Q S L Y S I X A A G
(b)
(c)
Ramachandran Plot
Psi (degrees)
Phi (degrees)
180 135 90 45 0 -45 -90 -135
-180 -135 -90 -45 0 45 90 135 180
Plot statistics
Residues in most favoured regions [A,B,L] 384 93.7%
Residues in additional allowed regions [a,b,l,p] 26 6.3%
Residues in generously allowed regions [~a,~b,~l,~p] 0 0.0%
Residues in disallowed regions 0 0.0%
Number of non-glycine and non-proline residues 410 100.0%
Number of end-residues (excl. Gly and Pro) 2
Number of glycine residues (shown as triangles) 44
Number of proline residues 33
Total number of residues 489
(d)
Error value*
99%
95%
320 340 360 380 400 420 440 460 480
Residue # (window center)
Error value*
99%
95%
20 40 60 80 100 120 140 160 180 200 220 240 260 280 300
Residue # (window center)
(e)

Figure S4

(a)

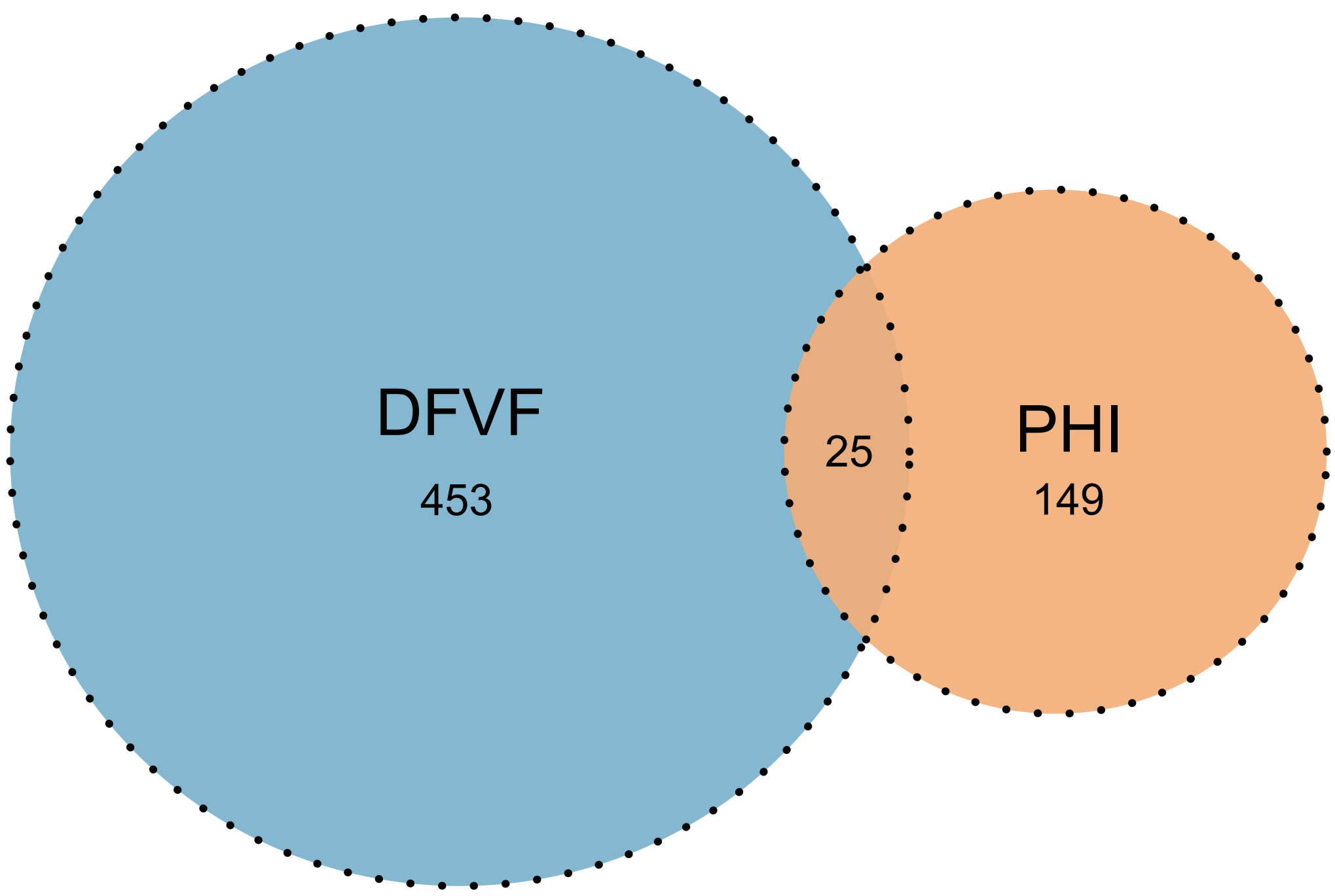

DFVF
453
25
PHI
149

(b)

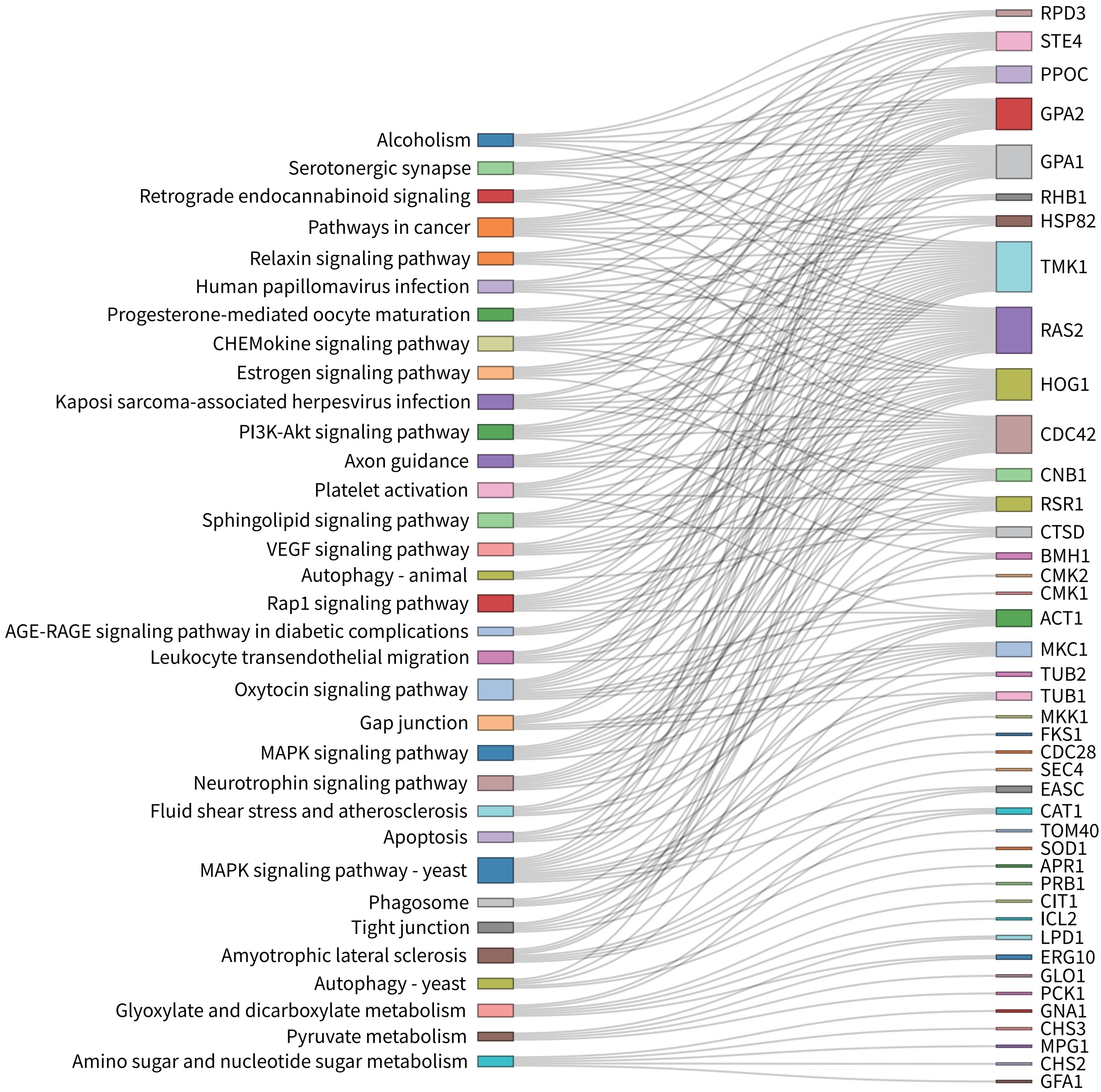

Alcoholism
Serotonergic synapse
Retrograde endocannabinoid signaling
Pathways in cancer
Relaxin signaling pathway
Human papillomavirus infection
Progesterone-mediated oocyte maturation
CHEMokine signaling pathway
Estrogen signaling pathway
Kaposi sarcoma-associated herpesvirus infection
PI3K-Akt signaling pathway
Axon guidance
Platelet activation
Sphingolipid signaling pathway
VEGF signaling pathway
Autophagy - animal
Rap1 signaling pathway
AGE-RAGE signaling pathway in diabetic complications
Leukocyte transendothelial migration
Oxytocin signaling pathway
Gap junction
MAPK signaling pathway
Neurotrophin signaling pathway
Fluid shear stress and atherosclerosis
Apoptosis
MAPK signaling pathway - yeast
Phagosome
Tight junction
Amyotrophic lateral sclerosis
Autophagy - yeast
Glyoxylate and dicarboxylate metabolism
Pyruvate metabolism
Amino sugar and nucleotide sugar metabolism
RPD3
STE4
PPOC
GPA2
GPA1
RHB1
HSP82
TMK1
RAS2
HOG1
CDC42
CNB1
RSR1
CTSD
BMH1
CMK2
CMK1
ACT1
MKC1
TUB2
TUB1
MKK1
FKS1
CDC28
SEC4
EASC
CAT1
TOM40
SOD1
APR1
PRB1
CIT1
ICL2
LPD1
ERG10
GLO1
PCK1
GNA1
CHS3
MPG1
CHS2
GFA1
